# Polyurethane Composite Foams Synthesized Using Bio-Polyols and Cellulose Filler

**DOI:** 10.3390/ma14133474

**Published:** 2021-06-22

**Authors:** Katarzyna Uram, Milena Leszczyńska, Aleksander Prociak, Anna Czajka, Michał Gloc, Michał K. Leszczyński, Sławomir Michałowski, Joanna Ryszkowska

**Affiliations:** 1 Faculty of Chemical Engineering and Technology, Cracow University of Technology, Warszawska 24, 31-155 Cracow, Poland; slawomir.michalowski@pk.edu.pl; 2 Faculty of Materials Science and Engineering, Warsaw University of Technology, Wołoska 141, 02-507 Warsaw, Poland; milena.leszczynska.dokt@pw.edu.pl (M.L.); anna.czajka2.dokt@pw.edu.pl (A.C.); michal.gloc@pw.edu.pl (M.G.); joanna.ryszkowska@pw.edu.pl (J.R.); 3 Faculty of Chemistry, Warsaw University of Technology, Noakowskiego 3, 00-664 Warsaw, Poland; mleszczynski@ch.pw.edu.pl; 4 Institute of Physical Chemistry, Polish Academy of Sciences, Kasprzaka 44/52, 01-224 Warsaw, Poland

**Keywords:** rapeseed oil-based polyol, microcellulose, rigid polyurethane foams, thermal conductivity, cell structure

## Abstract

Rigid polyurethane foams were obtained using two types of renewable raw materials: bio-polyols and a cellulose filler (ARBOCEL^®^ P 4000 X, JRS Rettenmaier, Rosenberg, Germany). A polyurethane system containing 40 wt.% of rapeseed oil-based polyols was modified with the cellulose filler in amounts of 1, 2, and 3 php (per hundred polyols). The cellulose was incorporated into the polyol premix as filler dispersion in a petrochemical polyol made using calenders. The cellulose filler was examined in terms of the degree of crystallinity using the powder X-ray diffraction PXRD -and the presence of bonds by means of the fourier transform infrared spectroscopy FT-IR. It was found that the addition of the cellulose filler increased the number of cells in the foams in both cross-sections—parallel and perpendicular to the direction of the foam growth—while reducing the sizes of those cells. Additionally, the foams had closed cell contents of more than 90% and initial thermal conductivity coefficients of 24.8 mW/m∙K. The insulation materials were dimensionally stable, especially at temperatures close to 0 °C, which qualifies them for use as insulation at low temperatures.

## 1. Introduction

Polyurethanes (PURs) are some of the most popular polymer groups in a $45 billion market. The cumulative annual growth rate in the market is projected to increase by 5% over the forecast period (2021–2026) [1]. PURs are produced in different forms, e.g., solid or porous, especially as foams of low apparent density. Rigid polyurethane foams (RPURFs) are quite popular thermal insulation materials, characterized by a low apparent density (10–70 kg/m^3^), high compressive strength, low brittleness, and low thermal conductivity. The initial thermal conductivity coefficient is mostly in the range of 19–24 mW/m·K, and such values are much lower compared to other popular commercially available materials, e.g., expanded polystyrene and mineral wool. Generally, the physical–mechanical properties of RPURFs are closely related to the characteristics of polyol components used, e.g., their molecular weight and functionality [2].

Conventional RPURFs are formed by a very exothermic reaction of isocyanates and polyols. The temperature inside the core of an expanded foam reaches 150–70 °C [3]. Current works on RPURFs are aimed at replacing petrochemical components with bio-based ones, improving foaming efficiency and reducing costs while maintaining the lowest possible values of the thermal conductivity coefficient. Due to environmental protection and awareness of the depletion of fossil fuels, there is a growing interest in the use of renewable raw materials for the production of PUR materials [4]. Therefore, there is a tendency to replace mainly petroleum polyol components with hydroxyl derivatives obtained from renewable raw materials [5]. An alternative to fossil fuels is vegetable oils, which can have a positive impact on the environment. Vegetable oils are available all over the world and are successfully used in the production of polyols, i.e., as one of the main components in the production of PURs [6]. Moreover, the prices of oils are lower than those of petrochemical raw materials [3,7]. Research concerning life cycle assessments for vegetable oil-based polyols showed a reduction of the fossil fuels consumption [8]. Various vegetable oils are used in the production of polyols, e.g., in Asia and America, palm oil and soybean oil, respectively. On the other hand, sunflower and rapeseed oils are the most popular in Europe [9,10,11]. This raw material consists of glycerol esters and higher fatty acids (saturated and unsaturated). Most vegetable oils, with the exception of castor oil, do not contain any hydroxyl groups in their chemical structures. Therefore, the oils must be chemically modified to introduce hydroxyl groups, which allow for the reaction with isocyanates to create urethane bonds. One of the most popular reactions for modifying unsaturated bonds (carbon-carbon double bonds) is the following two-step method: epoxidation and opening of oxirane rings [12]. In the first stage, unsaturated bonds are oxidized by using, for example, peracetic acid formed in situ in the reaction of acetic acid with hydrogen peroxide [13,14]. Subsequently, the epoxy rings are opened with compounds containing a reactive hydrogen atom, such as alcohols, amines, or water [15]. The properties of rigid polyurethane foams are closely related to those of the bio-polyols used to produce them. Therefore, the hydroxyl number and the distribution of hydroxyl groups in the bio-polyol backbone are important. The epoxidation/oxirane rings’ opening method gives primary and secondary hydroxyl groups in the bio-polyol. Secondary hydroxyl groups are less reactive with isocyanates than those containing primary hydroxyl groups and require longer curing times when reacting with isocyanates to form polyurethanes [16].

In the literature, there are studies demonstrating that a modification of PUR systems with bio-polyols has an influence on the properties of RPURFs. Kurańska et al. [17] modified a PUR system by replacing a petrochemical polyol with a rapeseed-oil-based bio-polyol. They observed that the reactivity of the PUR system decreased with increasing content of bio-polyol in the polyols premix. Increasing the proportion of the bio-polyol in the PUR system decreased the apparent density and the content of closed cells in the final RPURFs. Kirpluks et al. [18] synthesized RPURFs also with bio-polyols. The bio-polyols were obtained by opening oxirane rings with diethylene glycol and di- and triethanolamine. The resultant bio-polyols were then used for the production of RPURFs. The results showed that a low thermal conductivity coefficient in the range of 21–23 mW/m∙K is correlated to high closed-cell content, above 95%. Additionally, those foams achieved excellent mechanical properties, such as compressive strength above 0.20 MPa measured parallel to the direction of foaming.

Although bio-polyols are the main components that affect the properties of RPURFs, their properties can also be improved by introducing fillers [2,19]. In the literature, various RPURFs are described, in which fillers of various origins—nutmeg [20], egg shells [21], walnut shell [22], basalt powder [23], or fly ash [24]—were applied. The advantage of introducing natural fillers (like cellulose or lignin) is the presence of hydroxyl groups in their structures. Additionally, natural fillers can reduce the cost of foam production by partially replacing the polyol. This replacement can occur when the filler used has a linear or branched structure [2].

Cellulose is one of the most popular plant-derived polymer raw materials. As the chemical raw material, it is used in many everyday products and materials [25]. The use of this filler in the form of micro- or nanofibers increases the strength of the polymeric materials. This is due to the specific mechanical properties of individual nanocrystals [26]. Already a small amount of cellulose filler changes the morphology of rigid polyurethane foams by reducing the sizes of the cells [5].

Lignin is the most common chemical compound derived from wood. It is an integral part of the secondary cell walls of plants and some algae. X. Luo et al. [27] modified RPURFs with lignin originating from bioethanol production, up to 15 wt.%. Lignin is an amorphous polymer whose hydroxyl functional groups are active in hydrogen bonding. It was observed that the introduction of the filler increased the apparent density of the material from 62 to 86 kg/m^3^. The cell structure of the foams was more uniform with the addition of lignin and the cell walls became thinner. Moreover, the introduction of lignin up to 10% by weight increased the mechanical strength of the modified foams. Septevani et al. [28] prepared RPURFs with microcrystalline cellulose (0.2, 0.4, and 0.8 wt.%) in order to obtain materials with reduced thermal conductivity and improved mechanical properties. The decrease in the thermal conductivity was related to the decrease in the size of the cells and the increase in their number in the foam. The higher closed-cell content confirms that the filler had been incorporated into the PUR matrix without opening the foam cells. This effect was observed for the system modified with 0.4 wt.% of cellulose. In terms of the mechanical strength, no significant improvement was observed. Another work described the influence of melamine and silica as fillers on the properties of RPURFs [29]. The introduction of the fillers into the PUR system caused a reduction in the reactivity and problems with cell growth. Fillers reduce the reactivity of systems during the foaming process because the isocyanate and polyol molecules are less mobile, so the reaction is more difficult. Melamine and silica caused the nucleation of the molecules and the creation of nucleation sites for the gas phase. This led to a disturbance of the cell structure and cracking of the foam cell walls. This was confirmed by the results of compressive strength tests where, despite the higher apparent density of the materials, the strength was lower.

In this paper, a modification of RPURFs with two types of bio-polyols and a cellulose filler is presented. The bio-polyols used were obtained in a two-stage method of epoxidation and the opening of oxirane rings, while the filler was a microcellulose ARBOCEL^®^ P 4000 X. The foam recipe contained 40%wt. of the bio-polyols in the polyols mixture and various amounts of cellulose (1, 2, 3 php). The influence of the filler on the cell structure, apparent density and thermal conductivity of the modified foams was investigated. Additionally, changes in the compressive strength at 10% deformation and the thermal properties of the composites with cellulose were analyzed.

## 2. Materials and Methods

### 2.1. Materials

RPURFs were obtained from a petrochemical polyether polyol (Rokopol^®^ RF551, PCC, Rokita, Poland) and two types of bio-polyols from rapeseedoil. These bio-polyols were synthesized by a two-step process: epoxidation and opening of oxirane rings. As the oxirane ring opener, 1-hexanol and 1,6-hexanediol were used. The petrochemical polyol was replaced by bio-polyols at 40% by weight. The weight ratio of used bio-polyols was 1:1. The detailed procedure for obtaining bio-polyols was described in the previous work [30]. The characteristics of the polyols used are shown in Table 1.

Diphenylmethane diisocyanate (PMDI) with an isocyanate group content of 31% was used as the isocyanate component. A reactive amine catalyst (Polycat 218) for a strong urea reaction from Evonik Industries AG (Essen, Germany) and Surfactant Niax silicone (L-6915) from Momentive Performance Materials (Waterford, NY, USA)enabled stabilization of rising foams. Carbon dioxide resulting from the reaction of isocyanate groups with water was used as a chemical blowing agent. ARBOCEL^®^ P 4000 X, based on a natural, water-insoluble microcrystalline cellulose in the form of dispersible white powder was supplied by JRS Rettenmaier. CAS number: 9004-34-6; average particle size: >10 µm; bulk density: 400–700 g/L (DIN EN ISO 60).

The formulation of RPURFs modified with the bio-polyols and microcellulose is shown in Table 2. Due to the water present in the bio-polyols and in the cellulose dispersion, the amount of added water was respectively reduced (the total amount of water in all PUR systems was 3.5 g).

### 2.2. Preparation of Cellulose Dispersion in Petrochemical Polyol

ARBOCEL^®^ P 4000 X was dispersed in polyol Rokopol^®^ RF551 (6 php) in the following way: in the first stage, cellulose was dispersed in the polyol with a mechanical stirrer (10 min, 2000 rpm), and next, the system was calendered using three roll mills EXAKT 80T (EXAKT Advanced Technologies, Norderstedt, Germany) with a roll spacing of 100, 50, and 20 µm, at a speed of 30 to 90 rpm.

### 2.3. Characterization of Cellulose

The crystalline structure of a cellulose sample was investigated at room temperature by powder X-ray diffraction (PXRD) using a PANalytical Empyrean diffractometer (Almelo, the Netherlands) equipped with a Ni-filtered Cu Kα radiation source operating at 40 kV and 40 mA in the Bragg–Brentano geometry. The sample was scanned in the 2θ range 3–80° with a step size of 0.033° and a time per step of 60 s.

The morphology of the cellulose sample was analyzed using a high-resolution Scanning Electron Microscope (SEM) HITACHI SU8000, (Hitachi High-Technologies Corporation, Tokyo, Japan). The sample was initially deposited using a gold and palladium target for 150 s at 10 mA and 1.5 kV using a Polaron SC7640 (Quorum Technologies Ltd., Laughton, UK) sputter coater. 

The chemical structure of microcrystalline cellulose was examined using Nicolet 6700 (Thermo Electrone Corporation, Waltham, MA, USA). Spectra were recorded as a sum of 64 scans using a spectral range of 4000–400 cm^−1^.

The course of thermal degradation of microcrystalline cellulose was found through a thermogravimetric analysis (TGA) using the analyzer TGA Q500 TA Instruments, (Lukens Dr, New Castle, DE, USA). Sample of 10 ± 1 mg was tested in the nitrogen atmosphere and heated at a rate of 10 °C/min from room temperature to 800 °C. The result was analyzed using the Universal Analysis 2000 software (version 4.7) using a TA Instrument.

### 2.4. Preparation of Rigid Polyurethane Foams Modified by Microcellulose

RPURFs were prepared following the free-rise method. The petrochemical polyol and the bio-polyols were mixed with the catalyst, surfactant, water, and cellulose-polyol dispersion for 60 s. Systems containing 1, 2, and 3 php of cellulose were prepared. The appropriate amount of isocyanate was then introduced, and the reaction mixture was stirred again for 6 s and poured into a mould. The isocyanate index was 110. The RPURFs were left for 24 h at room temperature in order to cross-link the materials.

### 2.5. Characteristics of Rigid Polyurethane Foams

RPURFs modified with cellulose were tested to determine their physical, thermal, and mechanical properties.

The chemical composition of the PUR system was analyzed using absorption spectra obtained with a Fourier transform infrared (FTIR) spectrophotometer Nicolet 6700 (Thermo Electron Scientific, Waltham, MA, USA) with ATR (total reflection suppressed). Each sample was scanned 64 times in the 4000–400 cm^−1^ wavelength range. Analysis of the obtained spectra was carried out with the OMNIC 8.2.0.387 (Thermo Fisher Scientific Inc., Waltham, MA, USA) software.

The distributions of the filler particle size and cell size as well as total porosity were studied using an Xradia 400CT tomography device (Zeiss, Jena, Germany). The detailed measurements’ methodology was described in the article of Auguścik-Królikowska M. et al. [31].

The morphology of cells was analyzed using a SEM (Hitachi TM3000, Tokyo, Japan). An acceleration voltage of 5 keV was used. Before SEM observations, samples were deposited using the same sputter coater as the one described in Section 2.3—80 s at 10 mA and 2 kV. The anisotropy index was calculated as the ratio of the cell height to width.

The apparent density of the PUR foams was calculated from the ratio of the sample mass to volume. The samples were tested according to the ISO 845 standard [32].

The share of the closed-cell content was measured using the pycnometer method according to the ISO 4590 standard [33].

The thermal conductivity was tested using a Lasercomp Heat Flow Instrument Fox 200 (New Castle, DE, USA). The temperature difference between the plates was 20 °C. The test was carried out in accordance with the ISO 8301 standard [34]. The dimensions of the samples were about 200 × 200 × 50 mm^3^.

Compressive strength tests of the foams at 10% deformation were performed on a Zwick model Z005 TH Allround-Line (Ulm, Germany)machine at a compression speed of 2 mm/min. The tests were carried out in both directions of the foam growth: parallel and perpendicular. The tests were conducted in accordance with the 826:2013-07standard [35].

The dimensional stability was determined according to ISO 2796-1986 [36]. The measurement was carried out at −25 and 70 °C and 90% humidity for 24 h. RPURF samples with dimensions of 200 × 200 × 50 mm^3^ were used for the tests.

The course of thermal degradation was found through a thermogravimetric analysis (TGA) using the analyzer TGA Q500 TA Instruments, Lukens Dr, New Castle, DE, USA. Samples of 10 ± 1 mg were tested in the nitrogen atmosphere and heated at a rate of 10 °C/min from room temperature to 800 °C. The results were analyzed using the Universal Analysis 2000 software (version 4.7) using a TA Instrument.

The changes taking place in the materials as a function of temperature were measured using differential scanning calorimetry (DSC) Q1000 TA Instruments, Lukens Dr, New Castle, DE, USA. Measurements were taken in a helium atmosphere in hermetic aluminum pans. Samples of approximately 6 mg were heated at a rate of 10 °C/min from −80 to 250 °C.

## 3. Results and Discussion

### 3.1. ARBOCEL^®^ P 4000 X Characterization

**Figure 1:**
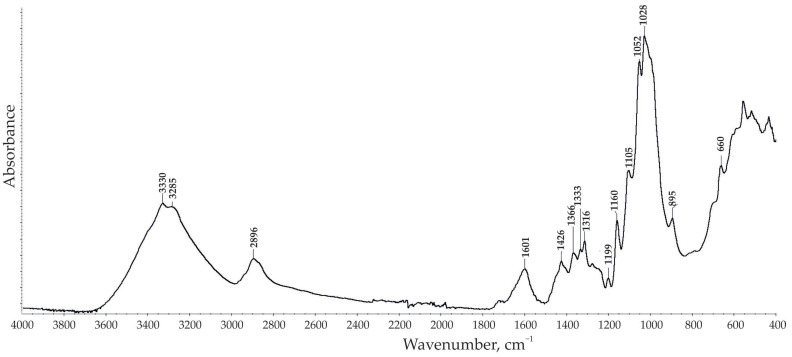
FTIR (Fourier Transform Infrared Spectroscopy) spectrum of microcellulose.

The FTIR spectrum of ARBOCEL^®^ P 4000 X is shown in Figure 1. The wavenumbers of the peaks are summarized in Table 3. The filler FTIR spectrum shows bands typical of cellulose compounds.

**Figure 2:**
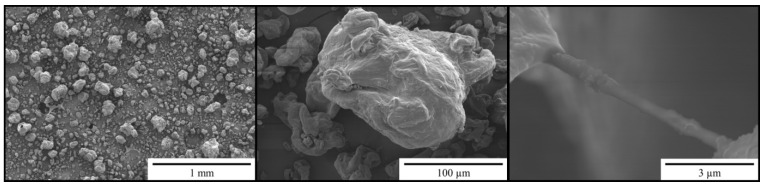
SEM (Scanning Electron Microscopy) images of ARBOCEL^®^ P 4000 X.

Figure 2 shows SEM images of microcellulose at different magnifications. The SEM images show particles with a size of 10–200 µm, as well as single fibers with a size >500 nm.

**Figure 3:**
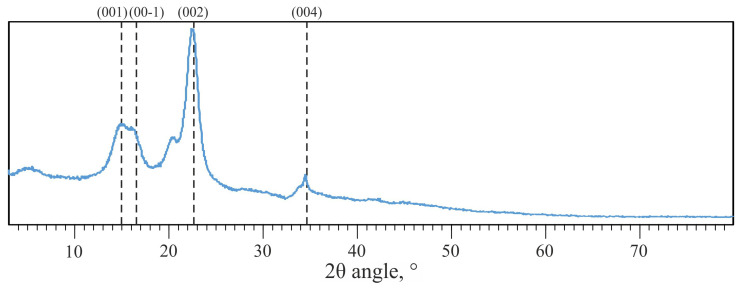
PXRD (Powder X-Ray Diffraction) diffractogram of ARBOCEL^®^ P 4000 X.

PXRD diffractogram of ARBOCEL^®^ P 4000 X is shown in Figure 3.

The cellulose sample was characterised by a crystallinity index (CI) of 74%, deter-mined using the peak height method in accordance with the literature data [40].

**Figure 4:**
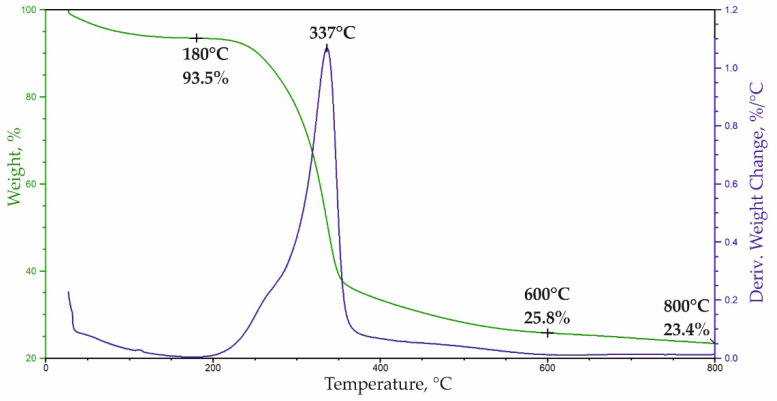
TGA analysis of ARBOCEL^®^ P 4000 X.

The degradation course of ARBOCEL^®^ P 4000X was assessed using TGA analysis (Figure 4). Based on the mass change curve (TG), the first stage of the decomposition fin-ished at 180 °C, while the second stage ended at 600 °C. In the first stage of the degrada-tion, about 6.5% of the mass was lost, which is related to the evaporation of water and oth-er volatile products that are found in ARBOCEL^®^ P 4000X.

**Figure 5:**
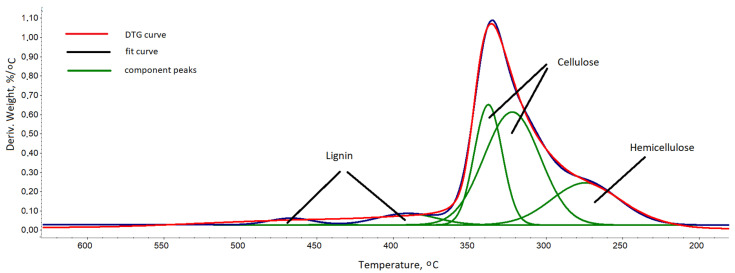
Deconvolution of ARBOCEL^®^ P 4000 X multiplet peak from DTG analysis into component peaks.

In the second stage of the degradation in the range of 180–600 °C, there was a loss of approx. 67.7% of the mass, which corresponds to the degradation of lignocellulose. After the degradation at 800 °C, approx. 23.4% of ash remained. The course of the lignocellulose degradation is represented by the multiplet peak on the derivate weight (DTG) curve in the range of 180–600 °C with a maximum at 337 °C. To evaluate the composition of lignocel-lulose, this multiplet peak was decomposed into component peaks (Figure 5) using a Gaussian distribution in the OMNIC 8.2.0.387 Thermo Fisher Scientific Inc. software.

Based on the half-area share of the component peaks, the proportion of primary com-ponents in ARBOCEL^®^ P 4000X was calculated, assuming that decompositions of hemi-cellulose, cellulose and lignin occur in the temperature ranges: 200–330, 250–370, and 350–600 °C, respectively. The authors of Vaisanen T. et al. [41] obtained similar results. It was found that the lignocellulose in ARBOCEL^®^ P 4000 X contains approx. 14% of hemi-cellulose, 49% of cellulose, and 5% of lignin.

### 3.2. Polyurethane Foams Characterization

**Figure 6:**
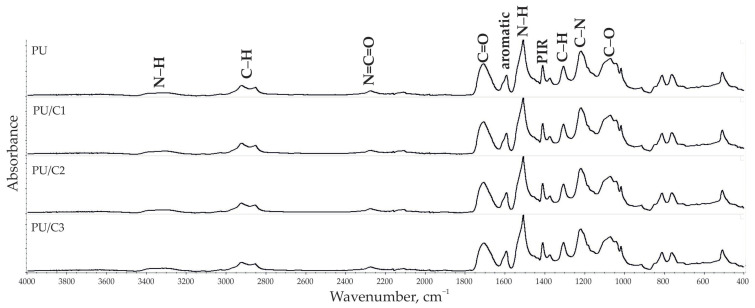
FTIR spectra of PUR foam and its composites with 1 php cellulose (PU/C1), 2 php cellulose (PU/C2), and 3 php cellulose (PU/C3).

Based on the FTIR spectra, the functional chemical groups of the PUR and PUR composite foams were identified (Figure 6).

The spectra of the PUR composites were similar to those of the PUR foam. The presence of groups characteristic for PURs was confirmed by the observation of the main absorption bands such as N–H stretching (broad band in the range 3200–3500 cm^−1^), C=O stretching (peak max. 1709 cm^−1^), and N–H bending (peak max. 1508 cm^−1^) [42]. These results suggested that the use of cellulose as a filler in the PUR composites resulted in no change in the chemical structure of the composites.

**Figure 7:**
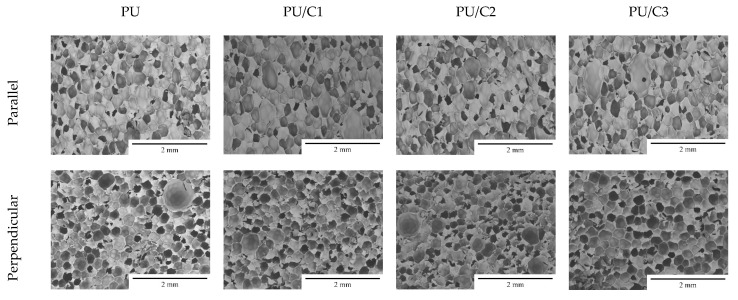
SEM images of PU, PU/C1, PU/C2, and PU/C3; magnification: ×40.

The cellular structure of RPURFs has a significant impact on their thermal insulation and mechanical properties. SEM images of the foam’s structure are shown in Figure 7, and the structure parameters are included in Table 4.

Introducing microcellulose in an amount of up to 3 php into the system did not change the shapes of the cells. The values of the anisotropy index in all the systems were similar. The anisotropy index is defined as the length to the width of the cell ratio. It is assumed that if the ratio is close to 1, the cell shape is almost like a sphere. The cells in the cross-section parallel to the foam growth direction were characterized by an anisotropy index higher than 1. This means that they have elongated shapes. The presence of such a cell shape is associated with the high reactivity of the PUR system. On the other hand, the cells in the cross-section perpendicular to the direction of the foam growth were almost spherical. Despite the lack of changes in the shapes of the cells, it was observed that the addition of cellulose to the PUR system increased the number of cells in the cross-section of the foam (parallel and perpendicular). The introduction of just 1 php of the filler had a significant impact on the number of cells in the RPURFs. It can be concluded that microcellulose gave a nucleation effect. This thesis is confirmed by the surface area of foam cells. In both cross-sections (parallel and perpendicular to the foam growth direction), the average cell surface areas of the filled foams were smaller than in case of the reference foam. The effect of increasing the number of cells after adding microcellulose or other fillers has been reported in the literature [5,43].

**Figure 8:**
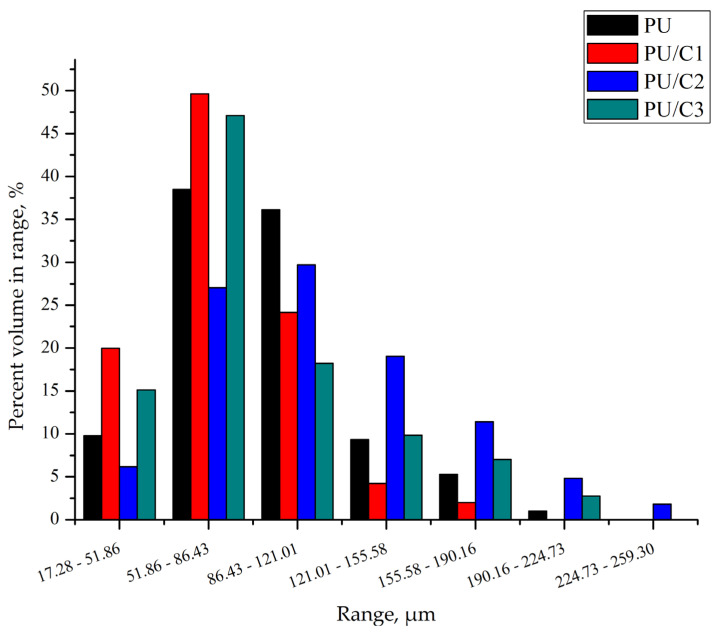
Distribution of pore size in PU foam and its composites with 1 php cellulose (PU/C1), 2 php cellulose (PU/C2), and 3 php cellulose (PU/C3).

Based on a computed microtomography study, it was found that the foams obtained in the experiments differed in terms of the pore size distribution, which is presented in Figure 8. The pore size of the foams was in the range of 20–260 µm. It was observed that approx. 75% of the pores in the PU foam are in the range 50–120 µm. In the composite containing 1 php cellulose, the pore size distribution is sharp with a clear indication of the maximum pore content (50%) corresponding to sizes in the range of 50–86 µm.

At 2 php cellulose, the pore distribution flattens out and is similar to the pore distribution in the PU foam; however, the pore size distribution in this material is narrower than in the other foams. The pore size distribution of PU/C3 foam is similar to that of the PU foam.

A similar reduction in the size of the foam cells after addition of 1% cellulose (CNC) was observed by Zhou et al. [43].

**Figure 9:**
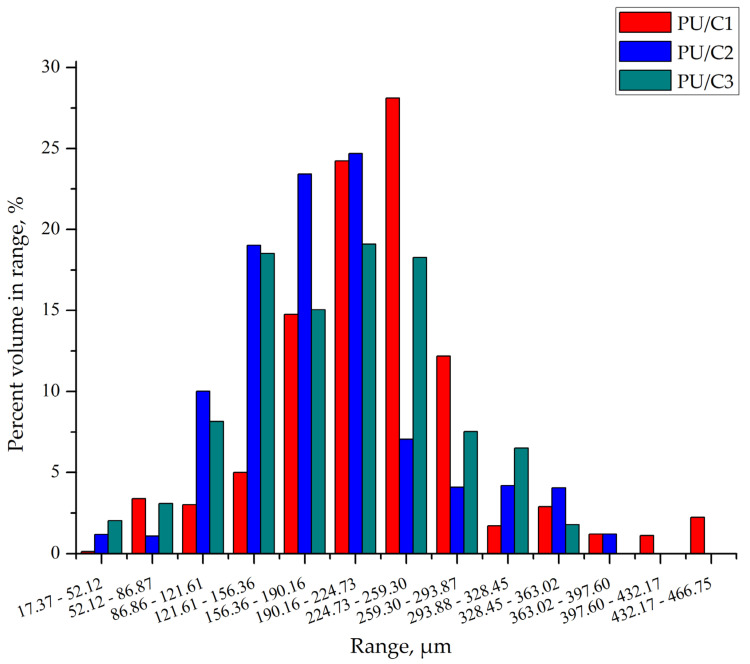
Particle size distribution in composites with 1 php cellulose (PU/C1), 2 php cellulose (PU/C2), and 3 php cellulose (PU/C3).

The sizes of the particles contained in the pore walls of the composites differed significantly (Figure 9). In the foam containing 1 php microcellulose, the particles and their agglomerates have sizes in the range of 17–470 µm, and 52% of them have sizes in the range of 190–260 µm. The character of the cellulose particle size distribution in the PU/C2 composite is similar to that of the PU/C1 composite. The largest particles in this foam are in the range of 156–224 µm and their share is approximately 48%. The particle size distribution of the PU/C3 foam is flatter than that of the other foams. In this foam, 71% of the particles are in the range of 120–260 µm.

The cell structure parameters are often in close correlation with selected physical-mechanical properties of RPURFs. Selected properties of the RPURFs obtained in our work are shown in Table 5.

Apparent density affects the thermal and mechanical properties of foams. The apparent density of the cellulose-modified systems was slightly lower than that of the reference material. The lowest values were obtained for the system modified with 2 php microcellulose. Increasing the share of microcellulose to 3 php resulted in a slight increase of the apparent density, but that value was still lower than in the case of the reference foam. Similar results were also obtained for RPURFs reinforced with industrial potato protein [44].

**Figure 10:**
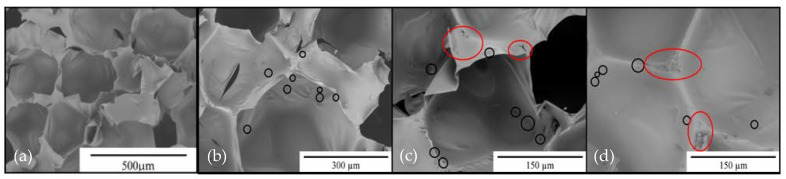
SEM images of (**a**) PU foam; (**b**)PU/C1; (**c**) PU/C2; and (**d**) PU/C3 foams.

Based on the analysis of µCT images, the total porosity (volume fraction of porosity in samples) in the foams was determined. The results are summarized in Table 5. The PU foam had the largest total volume of pores, while the cellulose foams had a lower total porosity than the PU foam. The results of the studies of the total porosity and apparent density indicate that in the foams containing microcellulose, there was a significant number of micropores with sizes below 17 µm, which were undetectable using the Xradia 400CT tomograph due to the detection and resolution limitations of the device. Consequently, the foam material PU/C2 was characterized with the lowest apparent density as well as the highest share of micropores among all the materials (Figure 10), despite having the lowest total porosity determined in the microtomography study.

In Figure 10a–d, the micropores are marked with black ovals, and in Figure 10c,d, microcellulose agglomerates are marked with red ovals. Around the cellulose agglomerates, there are micropores that are formed as a result of a reaction with water, during which CO_2_ is formed [45].

Thermal conductivity is a parameter that characterizes insulating materials. Its value is influenced by the apparent density, thickness of the insulating layer, and content of closed cells. The introduction of the microcellulose into the PUR system did not significantly affect the values of the thermal conductivity coefficient, which were in the range of 24.7–24.8 mW/m∙K. The lowest value was observed for the foam with 1 php cellulose, which was characterized by the highest content of closed cells. Lower values of thermal conductivity can also be associated with a lower values of apparent density and a reduced heat conductivity through the polymer matrix. The effect of the influence of the RPURF cell structure was observed in studies in which foam materials were obtained with dyes [46]. In the described case, the introduction of a filler into the polyurethane system reduced the size of the cells and reduced the heat transfer through the cell walls of the PUR foams.

Additionally, the thermal conductivity of RPURFs was tested 7 days after their preparation. It was observed that the thermal conductivity coefficient increased by 30%. In the literature, thermal conductivity is presented as the sum of the energy shares transferred by the gas filling the cells, the polymer matrix, and radiation [47]. Moreover, the share of the gas thermal conductivity in the foam cells is 60–80% and depends on the type of blowing agent used. The thermal conductivity of carbon dioxide formed by the reaction of water as a chemical blowing agent with an isocyanate is lower than that of air. During the seasoning of the material, gases in the foam cells are converted from carbon dioxide to air, which increases the thermal conductivity of the RPURFs. A similar effect was also shown in [48].

**Figure 11:**
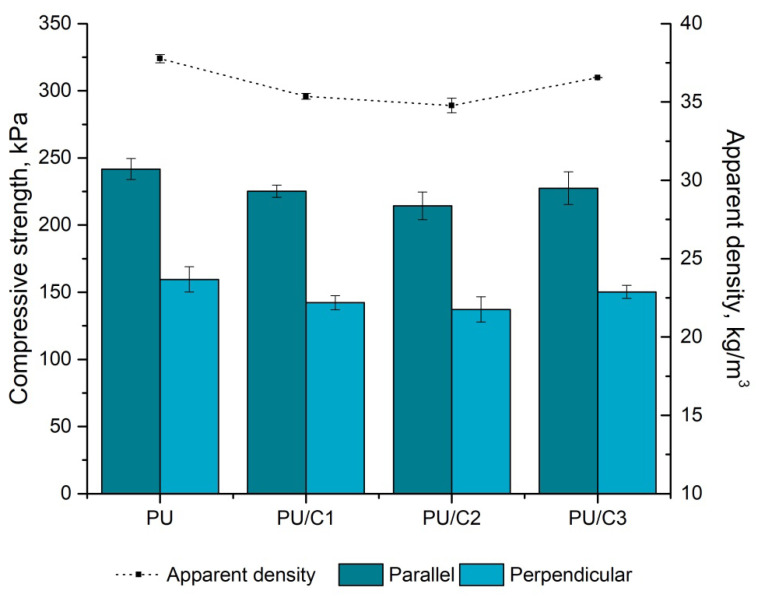
Changes in compressive strength and apparent density of PU foams with microcellulose.

The mechanical properties of cellulose-modified RPURFs were found by measuring the mechanical strength at 10% deformation. The strength values were closely related to the apparent density of the materials. Figure 11 shows the changes in the compressive strength depending on the microcellulose content in the PUR systems.

The anisotropic nature of the foam cells affects the mechanical properties of the material. The elongation of the cells in the direction of foam growth causes the compressive strength in the parallel direction to be higher than in the direction perpendicular to the foam growth direction. However, the compressive strength changes, as does the apparent density of the foam. A similar correlation was also described in the literature [49]. RPURFs with higher values of the apparent density are characterized by a higher cross-linking density of the material as well as by a higher stiffness. The material becomes more resistant to external forces [50]. The modification of the PUR systems with cellulose did not improve the compressive strength, but the values for the direction parallel to the direction of foam growth exceed 200 kPa. 

**Figure 12:**
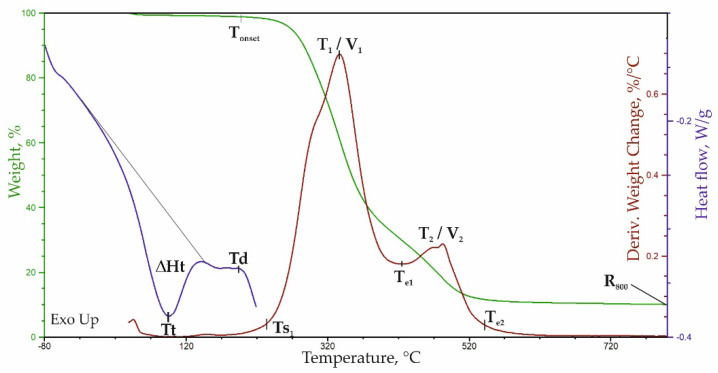
Curves of TG, DTG, and DSC analysis of PU foam.

**Figure 13:**
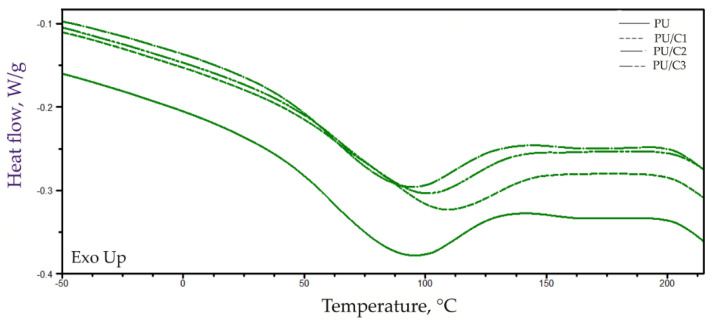
Curves of DSC analysis of PU foams in the first cycle analysis.

The TG, DTG, and DSC curves of the PUR foams are presented in Figure 12 and Figure 13.

On the basis of the DSC thermograms (Figure 12 and Figure 13) obtained in the first heating cycle, the parameters, such as order–disorder transformation temperature (T_t_), transformation enthalpy (ΔH_t_), and the temperature of the beginning of the degradation process (T_d_), were determined for all of the foams. The order–disorder transformation took place in the area of the hard phase of the PURs composed of segments containing urea and urethane groups. The temperature of this transformation was in the range of 85–98 °C (Table 6). The enthalpy of this transformation varied in the range of 33–39 J/g. The introduction of 1 php of the microcellulose resulted in an evident decrease of the order–disorder transformation enthalpy, while an opposite effect was observed upon the introduction of more than 1 php of the filler.

These results suggest that a small amount of microcellulose limits the ability to organize the rigid segments. The filler is located between the macromolecules, which limits the possibility of creating hydrogen bonds between the rigid segments. However, since the filler material contains some amount of water, an introduction of larger amounts of the filler leads to the creation of bonds between cellulose particles and NCO groups. More urea bonds are formed, which generates more hydrogen bonds connecting the rigid segments.

Moreover, a tendency for the degradation onset temperature (T_d_) to increase with an increasing amount of the filler in the composites was observed (Table 5). This change may be due to the increase in the number of urea groups in the composites.

From the TG and DTG thermograms (Figure 12), the following parameters were determined: the temperature of the onset of the degradation and the temperature of a 5% mass loss (T_5%_), the residue at a temperature of 800 °C (P_800_), the temperature at which the maximum rate of the degradation of the subsequent stages of the decomposition is T_1_/V_1_ and T_2_/V_2_, the temperature of the onset of the degradation at this stage (T_s1_), the temperature of the end of the first and second stages (T_e1_; T_e2_), and the mass change in stages 1 and 2 (Δm1 and Δm2) (Table 7).

The onset of the degradation determined from the DTG curve is approximately 201 °C. This result is in agreement with the result of the DSC analysis. On the basis of the data obtained during the TGA analysis, no differences in the course of the degradation of the composites can be noticed, which confirms the results of the FTIR analysis showing that the foam and its composites with the cellulose filler do not differ in terms of their chemical structures.

Another important parameter characterizing foam materials is the stability of linear dimensions, which was studied for all our foams (Table 8).

The tests were carried out in extreme conditions (at −25 and 70 °C and 90% humidity). In both cases of different measurement conditions, the changes of linear dimensions did not exceed 1%, and more favourable results were obtained for samples tested at temperatures below 0 °C.

## 4. Conclusions

Our results show that it is possible to obtain high-quality rigid polyurethane foams modified with both bio-polyols and microcellulose. The introduction of a cellulose filler caused cell nucleation and changed the cell structures of the rigid polyurethane foams. This effect was confirmed by an increase in the cell densities and a reduction of the cell sizes. Despite the changes in the sizes of the cells, their shapes did not change (the anisotropy indices of the two foams were 1.31 and 0.94, respectively). The rigid polyurethane foams were characterized by closed-cell contents of more than 90% and thermal conductivity coefficients of approx. 24.8 mW/m·K. Additionally, the compressive strengths of the foam materials were tested, and it was observed that the values in a direction parallel to the direction of the foam growth were over 200 kPa. Moreover, based on TG tests, it was found that the degradation of the foam begins at 201 °C and corresponds with the values determined in the DSC test. The insulation materials were dimensionally stable, especially at temperatures below 0 °C, which makes it possible to use them as insulation at low temperatures.

## Figures and Tables

**Table 1:** Properties of polyols.

Properties	Rokopol RF 551	Bio-Polyol BP-1.6HEX	Bio-Polyol BP-1HEX
Hydroxyl value (mgKOH/g)	420	217	101
Viscosity (mPa·s)	4000	2050	561
Water content (%)	0.1	0.25	0.04
Functionality	4.8	4.4	2.5

**Table 2:** Polyurethane systems with microcellulose.

Component (g)	PU	PU/C1	PU/C2	PU/C3
**Rokopol RF 551**	60.00	43.33	26.67	10.00
**Bio-polyol BP-1.6HEX**	20.00			
**Bio-poliol BP-1HEX**	20.00			
**Niax L6915**	1.50			
**Polycat 218**	1.50			
**Water**	3.37	2.87	2.37	1.87
**cellulose dispersion in Rokopol RF 551 polyol,6 php**	-	17.67	35.33	53.00
**PMDI**	141.93			

**Table 3:** Identification of peaks in FTIR spectrum [37,38,39].

Functional Groups	Wavenumber (cm^−1^)
OH stretching hydrogen bond	3330, 3285
C–H stretching	2896
C=C stretching	1601
C–H and H–C–H in-plane bending vibration	1426
C–H deformation vibration	1366
C–H or –OH	1333, 1316
C–O stretching vibrations	1199
C–O–C glycoside ether band	1160
C–O stretching vibrations	1105
C–O stretching vibrations	1055
C–O–C pyranose ring stretching vibration	1028
C–O–C, C–C–O, and C–C–H deformation modes and stretching vibrations in which the motions of the C-5 and C-6 atoms	895
C–O–H	660

**Table 4:** Cell morphology of PUR foams and their composites.

Foam System	Direction of Growth	Anisotropy Index	Number of Cell/mm^2^	Average Cell Cross-Sectional Area (mm^2^·10^3^)	N_F_^1^ (Number of Cells∙10^3^/cm^3^)
**PU**	Parallel	1.31 ± 0.04	45 ± 3	9.3 ± 0.7	301.3 ± 29.2
	Perpendicular	0.94 ± 0.04	70 ± 6	5.7 ± 0.4	592.4 ± 77.3
**PU/C1**	Parallel	1.31 ± 0.04	58 ± 2	7.3 ± 0.3	445.7 ± 27.3
	Perpendicular	0.95 ± 0.03	90 ± 6	4.6 ± 0.4	857.8 ± 77.8
**PU/C2**	Parallel	1.29 ± 0.07	60 ± 5	7.1 ± 1.0	460.5 ± 58.4
	Perpendicular	0.95 ± 0.04	91 ± 7	4.5 ± 0.6	863.4 ± 102.0
**PU/C3**	Parallel	1.31 ± 0.05	60 ± 4	7.4 ± 0.7	461.9 ± 42.4
	Perpendicular	0.94 ± 0.04	102 ± 5	3.9 ± 0.3	1027.0 ± 71.5

^1^ NF—cell density.

**Table 5:** Total porosity, apparent density, and thermal conductivity of RPURFs.

Foam System	Total Porosity (%)	Apparent Density (kg/m^3^)	Closed Cell Content (%)	Thermal Conductivity (mW/m·K)	
				(24 h)	(7 days)
**PU**	82.3	37.8 ± 0.27	91.0 ± 1.0	24.7 ± 0.08	31.2 ± 0.28
**PU/C1**	70.7	35.4 ± 0.19	92.2 ± 1.1	24.6 ± 0.03	31.6 ± 0.28
**PU/C2**	65.3	34.8 ± 0.47	92.2 ± 1.4	24.8 ± 0.19	32.5 ± 0.41
**PU/C3**	75.8	36.6 ± 0.01	90.7 ± 4.3	24.8 ± 0.16	32.5 ± 0.31

**Table 6:** Summary of DSC analysis results.

Sample/ Parameter	PU	PU/C1	PU/C2	PU/C3
**T_t_ (°C)**	91.0 ± 4.0	85.5 ± 10.0	90.7 ± 0.9	97.5 ± 0.8
**ΔH_t_ (J/g)**	38.8 ± 1.0	33.2 ± 2.0	35.5 ± 0.9	37.4 ± 1.5
**T_d_ (°C)**	202.7 ± 0.3	202.6 ± 0.2	203.2 ± 0.3	204.2 ± 0.4

**Table 7:** Summary of TGA analysis results.

Sample/ Parameter	T_onset_ (°C)	T_5%_ (°C)	T_s1_ (°C)	T_1_ (°C)/ V_1_ (%/°C)	T_e1_ (°C)	T2 (°C)/ V2 (%/°C)	Δm_1_ (%)	T_e2_ (°C)	Δm_2_ (%)	R_800_ (%)
**PU**	201	269	249	337/0.70	405	483/0.23	64.0	532	21.5	10.1
**PU/C1**	200	268	243	337/0.71	404	479/0.23	64.0	532	21.5	10.3
**PU/C2**	201	267	243	337/0.71	405	479/0.24	64.9	532	21.6	10.0
**PU/C3**	202	269	247	336/0.70	403	483/0.23	63.5	531	21.8	10.3

**Table 8:** Dimensional stability of RPURFs.

Foam System	Temperature: −25 °C			Temperature: 70 °C Humidity: 90%		
	Height	Width	Thickness	Height	Width	Thickness
**PU**	0.02 ± 0.08	0.02 ± 0.03	−0.12 ± 0.19	0.67 ± 0.07	0.70 ± 0.07	0.34 ± 0.25
**PU/C1**	0.06 ± 0.07	0.04 ± 0.07	0.44 ± 0.82	0.75 ± 0.09	0.77 ± 0.06	0.14 ± 0.16
**PU/C2**	−0.07 ± 0.06	−0.01 ± 0.05	0.05 ± 0.11	0.74 ± 0.15	0.77 ± 0.08	0.08 ± 0.25
**PU/C3**	0.02 ± 0.04	−0.05 ± 0.07	−0.05 ± 0.25	0.67 ± 0.09	0.68 ± 0.09	0.22 ± 0.12

## Data Availability

Data is contained within the article.
